# Drug Resistant Clinical Isolates of *Mycobacterium tuberculosis* from Different Genotypes Exhibit Differential Host Responses in THP-1 Cells

**DOI:** 10.1371/journal.pone.0062966

**Published:** 2013-05-07

**Authors:** Pampi Chakraborty, Savita Kulkarni, Ramakrishna Rajan, Krishna Sainis

**Affiliations:** Radiation Medicine Centre, Bio-Medical Group, Bhabha Atomic Research Centre, Mumbai, India; McGill University, Canada

## Abstract

*Mycobacterium tuberculosis* (MTB) persistently infects and survives within the host macrophages. Substantial genotypic variation exists among MTB strains which correlate with their interactions with the host. The present study was designed to establish a correlation, if any, between infection and induction of innate immune response by genetically diverse drug resistant MTB isolates from India. For this purpose, three clinical isolates from ancient and modern lineages, along with H37Ra and H37Rv were evaluated for intracellular growth, phagocytic index, induction of proinflammatory cytokines and apoptosis following infection in THP-1 cell line. A wide variation in the induction of cytokines was revealed subsequent to infection with different strains. EAI-5 strain from ancient lineage 1, induced higher proinflammatory responses, higher apoptosis and moderate intracellular growth compared to other strains, in contrast, for Beijing strain of modern lineage 2, all three parameters were lowest among the clinical isolates. Further, the responses induced by LAM-6 from modern lineage 4 were at a moderate level, similar to the laboratory strain H37Rv which also belongs to lineage 4. Thus, these profiles were specific to their respective lineages and/or genotypes and independent of their drug resistance status. Further, a positive correlation, among TNF-α, IL-1β, IL-6 and IL-12 induced in infected THP-1 cells was demonstrated. In addition, induction of all pro-inflammatory cytokines correlated well with the host cell apoptosis. A positive correlation was observed between phagocytic index in the category of ‘>10 bacilli/cell’ and induction of apoptosis, only for virulent strains, indicating that initial accumulation of MTB strains inside the host cell may be an important determining factor for different innate responses.

## Introduction

Tuberculosis (TB), the most prevalent infectious disease in the world, causes 1.4 million deaths each year including nearly 3, 50,000 deaths in India [1]. The innate immune responses to *Mycobacterium tuberculosis* (MTB), a causative agent of TB, by macrophages and dendritic cells (DC) play a crucial role in the host defense [2], [3]. The induction of the immune responses depends on the complex interplay between the host and the pathogen which may contribute to variations in immunopathology and transmission of the disease. Susceptibility to TB has been linked to polymorphism of certain host genes e.g. MHC, TLR-2, Vitamin D receptor, IFN-γ, IL-12R etc. [4], [5], [6]. It was initially believed that MTB complex constituted a genetically highly conserved group of bacteria, hence most of the earlier immunological studies have used a limited number of laboratory strains, such as H37Ra, H37Rv, Erdman and CDC1551 [7], [8]. The diversity in MTB genome, especially in the human-adapted strains, was demonstrated by evaluating polymorphisms at insertion elements, spacer elements in the direct repeat region and mycobacterial interspersed repetitive unit [9], [10]. Comas and Gagneux, demonstrated that MTB could be grouped into six main lineages and 15 sub-lineages using large sequence polymorphisms (LSPs) and these were named according to their geographical distribution [11]. It was also reported that lineages 1, 5 and 6 were ancient and; 2, 3 and 4 were modern on the basis of TbD1 analysis.

The genetically diverse MTB strains from different lineages have been shown to induce variable host responses in macrophages, cell lines and mouse models [12], [13], [14], [15], [16], [17], [18]. These strains are also known to vary with respect to their growth, virulence and immunopathology [19]. Selected W-Beijing strains elicited less proinflammatory and Th1 type cytokines than the non-W-Beijing strains. Further, Beijing and CAS1strains, belonging to lineage 2 and 3 respectively, showed lower growth rate and induced lower levels of proinflammatory cytokines in THP-1 cells [18] as well as macrophages from human PBMNC compared to standard laboratory strain H37Rv belonging to lineage 4 [20]. In contrast, another group detected higher induction of TNF-α by Beijing strain in human macrophages [14]. In a mouse model, genetically different MTB strains elicited dissimilar immune responses in lung, which determined differences in pathology and mortality. The Beijing genotype induced the highest mortality compared to H37Rv and Canetti genotype [13]. Further, apoptosis in the host cells may also contribute to innate host defense. Avirulent or attenuated strains were reported to induce significantly more apoptosis than virulent strains in alveolar macrophages [21]. Furthermore, host cell survival and apoptosis were also modulated by relative levels of TNF-α and IL-10 induced by MTB strains [22] and depended on phagocytic index for respective strains [23]. It is, therefore, important to evaluate phagocytosis and apoptosis in addition to intracellular growth and cytokines in the host cells infected with different MTB strains to get an explicit picture of the host defense in tuberculosis. Though there are several reports on host responses induced by MTB strains, no such studies on host response have been carried out with Indian MTB strains. Therefore, the present study was carried out to comprehensively evaluate all above mentioned parameters of host response in the monocyte leukemic cell line, THP-1, infected with well characterized drug resistant clinical isolates of EAI, Beijing and LAM genotypes from lineage 1, 2 and 4 of MTB, respectively, along with H37Ra and H37Rv. The correlation of these responses, if any, with the drug resistant status of the MTB strains was also assessed.

## Materials and Methods

### Characterization of M. tuberculosis Strains

Three clinical isolates used in the study were kindly provided by Department of Microbiology, KEM hospital and Tata Memorial Hospital, Parel, Mumbai and were selected on the basis of their spoligotyping pattern assessed using the kit from Isogen Bioscience B.V., Maarsen, Netherlands. Drug resistance status was evaluated by a susceptibility test performed as per the recommendations by WHO. After the documentation of a binary code, the spoligotypes were assigned as per the updated version of the international spoligotype database SpolDB4 [24]. EAI, Beijing and LAM strains from lineage 1, 2 and 4 respectively along with laboratory strains H37Ra and H37Rv were selected. DNA was extracted from all the strains by the standard cetyl-trimethyl ammonium bromide (CTAB) method [25]. In order to distinguish between modern and ancient genotypes, PCR was performed using TbD1 (MTB specific deletion 1) and RD1 specific primers [26]. In addition, MIRU-VNTR typing was performed by amplifying 12 MIRU-VNTR loci and the results were combined to form a 12-digit allele profile [10].

### Mycobacterial Growth and Single Cell Suspension

The MTB strains from patients were isolated on Lowenstein–Jensen (LJ) media (HIMEDIA, Mumbai) and after confirmation of a pure culture and biochemical tests, single colony was added to complete Middelbrook 7H9 medium (HIMEDIA) to get mid log phase culture. The cells were harvested at this point and stored in glycerol at −70°C. Before every infection experiment, these cell stocks were grown into log phase and used. The passage number was maintained at 5–6 for all the experiments. Single cell suspensions were prepared as per the standard protocol with minor modification [27]. Briefly, the cell pellets were washed, suspended in PBS containing 0.2% Tween 20 and transferred to a hard glass test tube containing around 25 glass beads (3 mm diameter). After bath sonication for 30 sec and vigorous vortexing for 5 minutes, the suspension was kept undisturbed for half an hour. The cell count was monitored by taking optical density (OD) of the upper cell layer at 600 nm and finally adjusted as required for infection experiments. The absence of clumps was confirmed by Ziehl–Neelsen Carbol Fuchsin (ZNCF) staining and the cell viability was evaluated by colony forming units (CFU) assay in each preparation.

### Host Cell Culture and Infection

As a genetically consistent model host, THP-1 cells were used in this study. THP-1 cell line was obtained from the National Center for Cell Science, Pune, India. It was maintained in RPMI 1640 medium (GIBCO, USA) supplemented with 2 mM L-glutamine, 10 mM HEPES buffer, 1.0 mM sodium pyruvate and 10% fetal bovine serum at 37°C in 5% CO_2_ humidified incubator. The cells were differentiated into macrophages by treatment with 20 nM phorbol-12-myristate-13-acetate (PMA, Sigma). After overnight incubation, the monolayer formed was co-cultured with different MTB strains at MOI (multiplicity of infection) of 10 (10∶1 bacilli/THP-1 cells), for 4 hours. The infected cells were washed three times with PBS to remove extracellular bacilli. Same passage number of THP-1 was maintained for study of responses to different MTB strains.

### Phagocytic Index

To study the phagocytic index, the THP-1 cells were seeded on a sterile glass coverslip and infected with MTB strains used in this study. After four hours of infection, the numbers of internalized bacilli were counted microscopically, using Ziehl Neelsen (ZN) acid fast and phenolic auramine staining. At least 300 consecutive macrophages were counted and grouped according to the number of intracellular bacteria.

### Assay of Intracellular Growth

To determine intracellular growth of various MTB strains, at different time points, CFU assay and a modified radiorespirometric assay were used [28]. Radiorespirometry works on the principle similar to BACTEC460 which uses ^14^C acetate and correlates well with CFU as well as spectrophotometric OD calculations [29]. In this experiment, monolayers of THP-1 cells were prepared in 24-well plates (10^5^cells/well) and then were infected with the MTB at MOI of 10. Infected cells were incubated for 4 h at 37°C in 5% CO_2_, and were washed three times with PBS to remove extracellular bacilli. Infected cells were further incubated in medium for 1 to 5 days. At the end of the incubation, cells were lysed with 1 ml of sterile distilled water. After 15 minutes, the lysis was confirmed by microscopic observation and the lysate was properly mixed. One hundred micro-litres of the lysate was transferred into radiorespirometry vial with 1 µCi ^14^C acetate from BRIT (Board of Radiation and Isotope Technology), Mumbai, India. Another aliquot of 100 µl was serially diluted and plated on complete 7H11 Middlebrook media agar plate in triplicate for evaluation of CFU. The vial used for radiorespirometry was an assembly of inner small vial containing LJ medium (without glycerol) and an outer vial having Whatman paper1 dipped in alkaline scintillation cocktail, forming a hemi cylinder. The paper was dipped in a mixture of liquifluor PPO-POPOP (2,5 diphenyloxazole-1,4-bis(5-phenyloxazoly) benzene) toluene concentrate and 4.0 N NaOH-methanol and dried before use. The generation of radioactive ^14^CO_2_ was determined daily with a Perkin Elmer Liquid Scintillation Analyzer (Tri-Carb 3100TR) and the data are presented as cumulative cpm (counts per minute). The cpm obtained on day 5 of the assay was compared for all MTB strains and were correlated with CFU.

### RNA Extraction, cDNA Synthesis and Real-time Reverse Transcription-polymerase Chain Reaction (RT-PCR)

RNA was extracted using a commercial RNA extraction kit (Qiagen) according to the manufacturer’s instructions. Thereafter, cDNA was synthesized using 1 µg of RNA by cDNA synthesis kit (Cat.No.#K1622, Fermentas Life Science). Quantitative real-time RT-PCR was performed with TNF-α, IL-1β, IL-12, and β-actin primers [30], [31] using SYBR Green master Mix (CAT # 600548, Stratagene, La Jolla, Ca, USA ) with the following amplification conditions: initial denaturation of 10 min at 95°C followed by 40 cycles of 95°C for 15s, 60°C for 30s and 72°C for 30s. Melting curve analysis was performed for confirming the specificity of PCR. Further, the Ct values for each gene amplification were normalized with respect to the house-keeping gene, β-actin by 2^–ΔΔCt^ method [32] and the expression levels are presented as fold induction in comparison to uninfected THP-1 cells.

### Cytokine Estimation by ELISA (Enzyme-linked Immunosorbent Assay)

Supernatants from infected cells (24 and 48 hours) were collected, centrifuged and frozen at −70°C until used. Determination of TNF-α, IL-1β, IL-6, IL-12 and IL-10 was carried out using commercial ELISA kits (BD OptEIA, Franklin Lakes, NJ, USA).

### Apoptosis Assays

#### Flowcytometric analysis

After 5 and 6 days of infection, THP-1 cells were washed with Annexin-binding buffer (10 mM HEPES; 0.14 M NaCl; 2.5 mM CaCl_2_; pH 7.4) and 5 µl of Annexin V-FITC (Sigma, USA) was added to 10^6^ cells. Cells were gently mixed and incubated for 15 min at room temperature in dark. Thereafter, the cells were acquired in a flowcytometer (Partec Cyflow Space, Görlitz, Germany) and analyzed by Flowmax software version 2.0.

#### Apoptosis ELISA

Apoptosis induction by different strains was measured by nucleosomal fragmentation ELISA (Cell Death Detection ELISAplus, Roche Applied Science, Indianapolis, IN). Briefly, 10^4^ cells were plated per well in 96-well plates, in presence and absence of MTB strains for 5 days and after removal of the supernatant, cells were lysed with the lysis buffer provided in the kit. After centrifugation of the lysate, supernatant was carefully transferred to precoated microtitre plate and subjected to ELISA as per the manufacturer’s protocol. The absorbance values were normalized to those from uninfected cells to derive an enrichment factor as per the manufacturer’s protocol (Roche Applied Science).

#### Western Blot

Western blot was performed as previously described [33]. In brief, infected THP-1cells were lysed, centrifuged and 80 µg of protein was resolved in 10% SDS-polyacrylamide gel for one hour at 100V. The proteins were transferred to a nitrocellulose membrane (Sigma) and after blocking for 2 hr, were incubated with rabbit antibodies against PARP (Poly ADP ribose polymerase) or β-actin followed by biotinylated anti-rabbit secondary antibody (all antibodies from Cell Signaling, Beverly, MA, USA). After washing, the membranes were developed with chemiluminescence reagents (Roche) and exposed to x-ray film.

### Statistical Analysis

Statistical analysis was performed using Sigmastat 3.5 and Microsoft Excel Statistical software. To determine differences between measurements from individual isolates ‘One Way Analysis of Variance’ was performed and P≤0.05 was considered as significant. For correlation between proinflammatory cytokine production, apoptosis, intracellular growth and phagocytosis, the ‘Pearson correlation test’ was performed and P≤0.05 was considered as significant.

## Results

### Characterization of M. tuberculosis Strains

Table-1 depicts the genotypic characteristics for the MTB strains selected for the study. The presence of intact TbD-1 region confirmed that EAI strain in the study was of an ancient lineage and its absence indicated that LAM and Beijing belonged to modern lineages. EAI-5 and LAM strain were resistant to eight and six drugs respectively whereas, Beijing strain was conventional XDR with resistance to ten drugs. A phylogenetic tree of isolates used in this study along with nearly 200 other well typed strains was constructed using spoligotype and 12-digit allele profile of MIRU (http://www.miru-vntrplus.org) [34] (Fig. S1).

**Table 1:** Genotypes of *M. tuberculosis* strains used in the study.

Clades	LineageNo.	Spoligocode	SIT*	MIRU-VNTR #	TbD-1	RD1
EAI5	1	777700777413700	763	254326223513	**+**	+
LAM6	4	777777607560771	64	123326153328	−	+
Beijing	2	000000000003771	1	222325153533	**−**	+

* SIT = Spoligo-International-Type number.

# 12 loci in the order = M2, M4, M10, M16, M20, M23, M24, M26, M27, M31, M39, M40.

### Phagocytic Index for Different Strains of MTB

**Figure 1:**
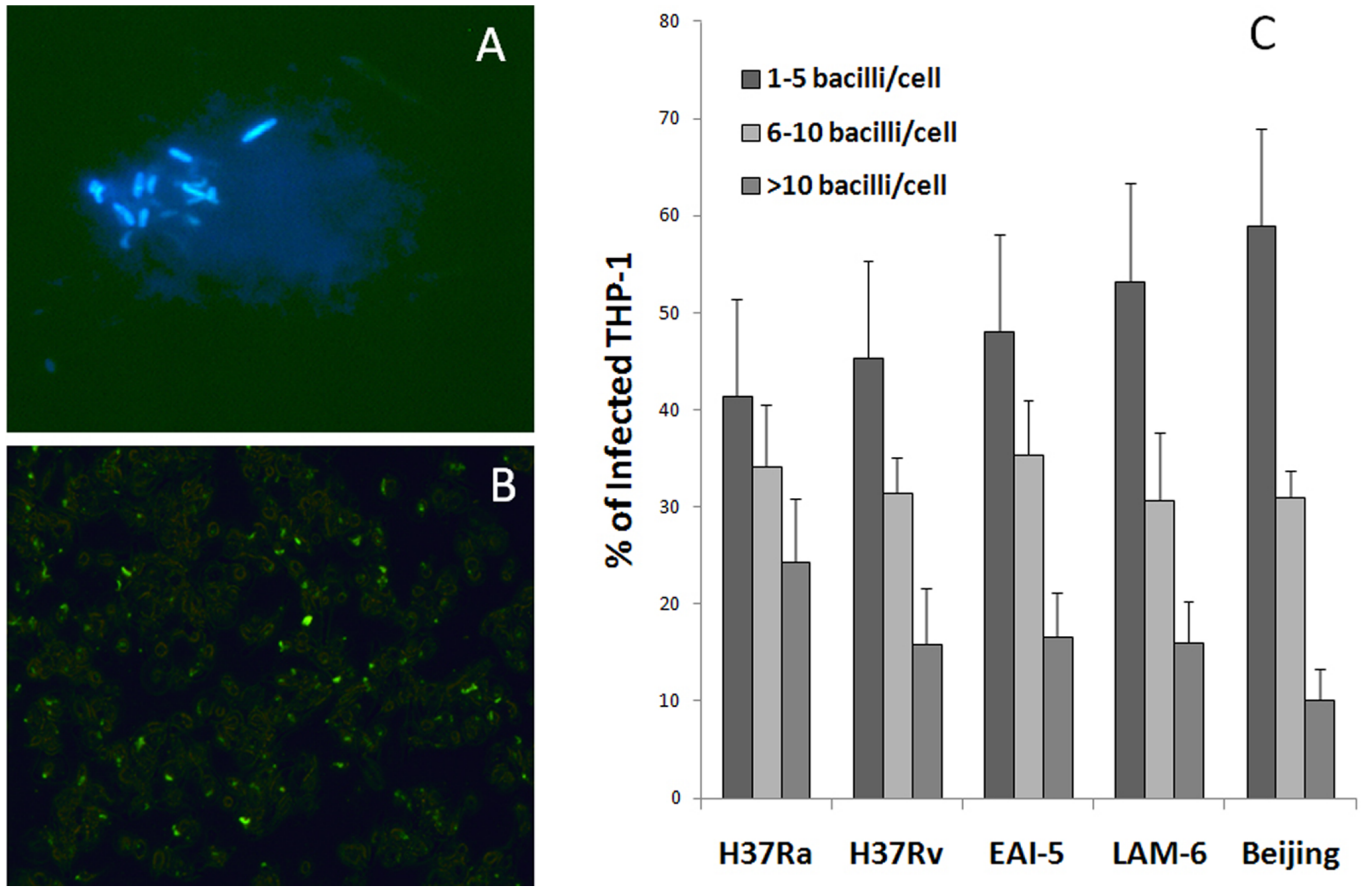
Phagocytosis of different strains of MTB by THP-1 cells. The representative photographs show THP-1 cells infected with MTB and stained with auramine-KMnO_4_ after 4 hours of infection, seen under UV light (Olympus, Tokyo) (A) at high magnification (×1000) and (B) low magnification respectively (×400). (C) Phagocytic index was categorized into 1–5 bacilli, 6–10 bacilli and >10 bacilli per cell. Each bar represents the mean ± SD for pooled values from five independent experiments.

The extent of infection by all MTB strains in THP-1 cells was scored on the basis of phagocytic index using phenolic auramine staining (Fig. 1A and 1B). All the strains showed comparable infectivity in THP-1 cells (65% to 70% after four hours of infection). However, when the cells showing phagocytosis were divided into three different groups viz. 1–5, 6–10 and greater than 10 bacilli per cell, it was found that significantly higher percentage of THP-1 cells infected with H37Ra, showed higher accumulation of bacteria i.e. 24±7% cells in the category ‘>10 bacilli/cell’ than those infected with either Beijing (10±3%) or LAM (15±4%) strains. The percentage of infected macrophages in ‘1–5 bacilli/cell’ category was higher compared to ‘6–10′ and ‘>10 bacilli/cell’ categories for all the strains (Fig. 1C).

### Assessment of Intracellular Mycobacterial Growth in Infected THP-1 Cells

**Figure 2:**
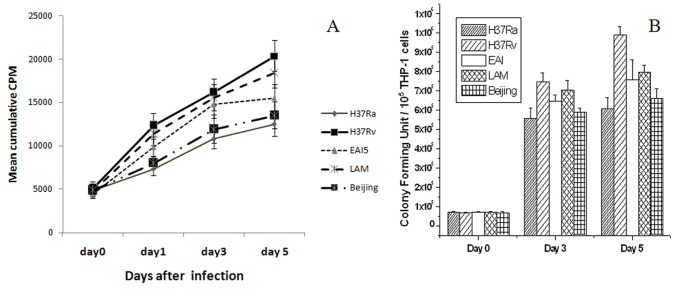
Intracellular growth of different MTB strains. THP-1 cells were infected with H37Ra, H37Rv, EAI, LAM and Beijing strains of MTB at MOI of 10 for 4 hrs and after removing extracellular bacteria, the infected cells were further incubated with medium for 1–5 days. After each incubation time, the infected cells were lysed. The lysates of infected cells were either inoculated in radiorespirometry vial containing LJ medium with ^14^C acetate or serially diluted and plated for CFU assay. For radiorespirometry (A), the counts were taken in a Liquid Scintillation counter (LSC) five days after vial preparation and for CFU assay (B) colonies were counted after 30 days of plating. Three such independent experiments were carried out and the data points represent mean ± SEM from all three experiments.

The intracellular growth was monitored by radiorespirometry and as CFU. Figure 2A shows the cumulative response (CPM obtained for ^14^CO_2_ released by viable bacilli), observed by radiorespirometry technique for intracellular bacterial load of different MTB strains at different time points. Fig. 2B shows CFU counts obtained for corresponding time points after infection. The radiorespirometry data correlated well with CFU counts (R^2^ = 0.98, P<0.05), confirming that the signal observed in radiorespirometry was from intracellular bacteria. A gradual increase in intracellular bacilli was observed for all the strains, with H37Rv and LAM-6 showing significantly higher intracellular bacillary growth compared to H37Ra. Though, THP-1 cells infected by different strains showed different percentages of cells in three different categories mentioned above, the total number of intracellular bacteria on day zero were in the range of 6.6 × 10^4^ to 7.1 × 10^4^ (as per CFU assay), which were not significantly different to give differences in cpm values in the radiorespirometry assay.

### Cytokine Profiles in THP-1 Cells after Infection with Different Strains

**Figure 3:**
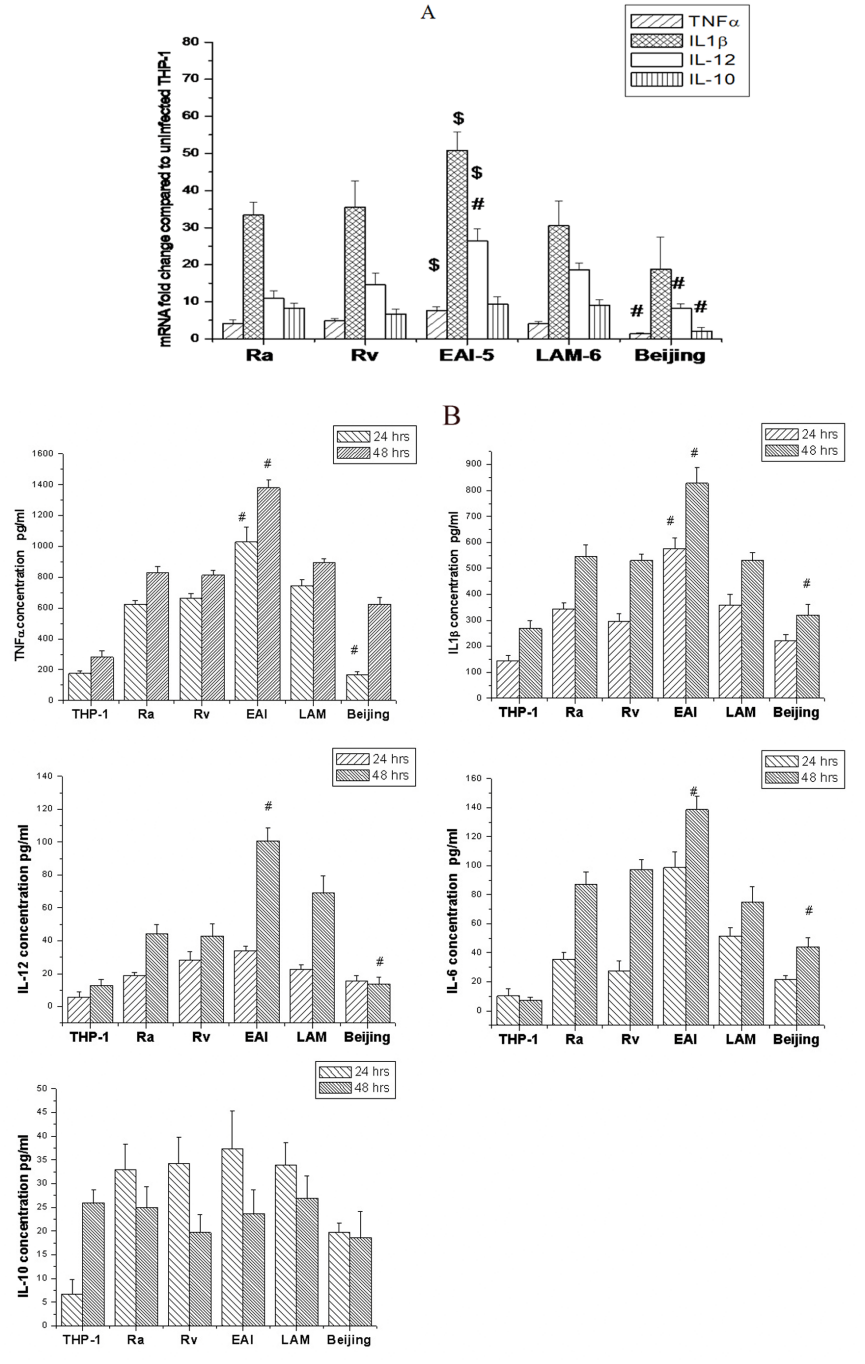
Cytokine induction in infected THP-1 cells. Real time PCR was carried out to estimate the mRNA expression for TNF-α, IL-1β, IL-12 and IL-10, 24 hrs after infection of THP-1 cells with *M. tuberculosis* H37Ra, H37Rv and three clinical isolates at MOI 10. The graph shows (A), relative mRNA expression levels presented as fold increase over specific mRNA obtained from uninfected THP-1 cells, after normalizing with housekeeping β-actin mRNA, using 2^–ΔΔCt^ method. Three independent experiments were carried out. Data represent the means ± SD of a representative experiment. #, compared to H37Rv and $, compared to Beijing strain. (B) Shows the levels of TNF-α, IL-1β, IL-12, IL-6 and IL-10 as measured by ELISA, in the supernatant of THP-1 cells infected with different strains of MTB, 24 and 48 hrs after infection. Three such independent experiments were carried out and data represent the mean ± SD of representative experiment. #, compared to H37Rv for respective time points.

The mRNA expression for proinflammatory cytokines like TNF-α, IL-1β, IL-12 and anti-inflammatory cytokine IL-10, induced by different MTB strains in infected THP-1 cells, showed variable patterns (Fig. 3A). The levels of different cytokines when measured by ELISA corroborated the mRNA expression patterns (Fig. 3B). The expression of TNF-α, IL-12 and IL-1β, both at mRNA and protein level, was significantly (P<0.05) higher in EAI-5 infected THP-1 cells than those infected with H37Rv, H37Ra and Beijing strains. However, the infection with Beijing genotype induced lower levels of all cytokines mentioned above. The induction of proinflammatory response by LAM-6 was comparable with that by H37Rv. Further, there was a significant correlation among proinflammatory cytokines, (TNF-α and IL-1β, R^2^ = 0.879, P<0.05; TNF-α and IL-6, R^2^ = 0.799, P<0.05; IL-1β and IL-6, R^2^ = 0.927, P<0.05). There was no significant difference in expression of IL-10 mRNA among the cells infected with different strains, except for Beijing which induced significantly lower mRNA for IL-10 compared to other strains (P<0.05). However, the protein concentrations of IL-10 in the supernatants of THP-1 cells, 24 and 48 hours of post-infection, were low with no significant differences among the strains. The most interesting observation about Beijing strain was that it induced lesser amount of both pro and anti-inflammatory cytokines in THP-1 cells in contrast to their levels in supernatants of cells infected with other strains.

### Apoptosis in Infected Cells

**Figure 4:**
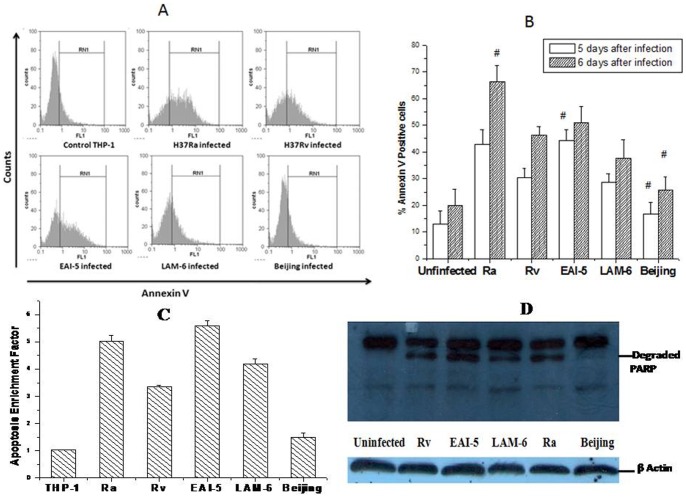
The apoptosis of infected THP-1 cells. (A) Flowcytometric profile of Annexin V-FITC labeled THP-1 cells infected with various MTB strains on 5th day of infection from a representative experiment of the three independent experiments. RN1 indicates the percentage of Annexin-V positive cells. (B) The bar diagram represents the percentage of infected THP-1 cells stained with AnnexinV after 5 and 6 days of infection. (C) Apoptosis was also determined using the Cell Death Detection ELISA (Roche Applied Science) after five days of infection. The bar diagram represents the extent of nucleosome fragmentation in infected THP-1 cells. (D) Expression of degraded PARP and β-Actin was monitored in THP-1 cells after 48 h of infection with five different MTB strains by Western Blotting. In this representative picture, the lanes were aligned from original picture.

Significant apoptosis was observed after 5 days of infection in THP-1 cells as seen from flowcytometric data in Fig. 4A. The extent of apoptosis induced by different strains in THP-1 cells assessed by Annexin V labeling (Fig. 4B) and apoptosis ELISA (Fig. 4C), was compared after five and six days of infection. Among the five strains, H37Ra and EAI-5 induced significantly higher apoptosis compared to that in uninfected cells and cells infected by Beijing strain. Further, the apoptotic response to LAM-6 was similar to that observed for H37Rv. The degradation profile of pro-apoptotic protein, PARP in uninfected and infected THP-1 cells after 48 hrs of infection is depicted in Figure 4D and also corroborates the extent of apoptosis.

### Correlation between Apoptosis, Pro-inflammatory Cytokines and Phagocytic Index

A good correlation was observed between levels of all proinflammatory cytokines and apoptosis induced only by virulent strains after five and six days of infection. The correlation coefficients (R^2^) for TNF-α, IL-1β, IL-6 and IL-12 with percent apoptosis were 0.906, 0.864, 0.879 and 0.890 respectively (P<0.05) on 5^th^ day and 0.830, 0.669, 0.639 and 0.846 respectively (P<0.05) on 6^th^ day of infection. When H37Ra was included for analysis, a significant correlation was not observed.

A positive correlation was observed between phagocytic index for ‘>10 bacilli/cell’ and the percent apoptosis after five (R^2^ = 0.708, P<0.05) and six (R^2^ = 0.608, P<0.05) days of infection and a negative correlation was seen between phagocytic index for ‘1-5 bacilli/cell’ and percent apoptosis after five (R^2^ = 0.6, P<0.05) and six (R^2^ = 0.606, P<0.05) days of infection, for all the strains.

## Discussion

Pathogenesis in tuberculosis is driven by many components of the host immune system, pathogen and environment [35]. The present study was focused on an integrative approach by evaluating different parameters of host-pathogen interactions using well characterized drug resistant MTB clinical isolates of different genotypes obtained from India.

The higher prevalence of Beijing and EAI spoligotypes which belong to Lineage 2 and Lineage 1 respectively in Indian subcontinent was well established earlier [36], [37]. In view of this, drug resistant strains representing ancient (EAI-5) and modern lineages (Beijing and LAM-6) were selected amongst the pool of clinical isolates. The strains from Beijing lineage are the most studied ones and are known for their drug resistance, hyper-virulence, relapse and global distribution [38], [39], [40]. The strains of LAM lineage are also known for causing cavitary disease [41] and the acquisition of drug resistance [42]. On the other hand, EAI belongs to a primitive subset which includes more drug sensitive strains, is hypothesized to be less virulent and is known for its higher prevalence in south India.

Innate immune mechanisms are crucial to the outcome of infection. In the present study, innate responses were comparatively evaluated for three MTB clinical isolates and two laboratory strains in terms of phagocytosis, intracellular growth, cytokines released and induction of apoptosis. The percentages of THP-1 cells showing phagocytosis were not significantly different for laboratory strains and clinical isolates. However, we observed that infection with avirulent H37Ra resulted in the higher number of infected cells with more than 10 mycobacteria per cell, compared to infection with all virulent strains which showed more infected cells in 1–5 mycobacteria per cell group. This is in agreement with the report of Rajavelu *et al* [23]. Other studies have compared the overall extent of phagocytosis for different MTB strains and have reported contradictory results. Torrells et al demonstrated that clinical isolates from Beijing genotype exhibited lesser phagocytosis due to the presence of truncated mannose-capped lipoarabinomannan (ManLam) as compared to laboratory strains [43] whereas, Sarkar et al have demonstrated less uptake of laboratory strain H37Rv as compared to that of clinical isolates at the time of infection [44]. It is now known that virulent strains of MTB are phagocytosed specifically via mannose receptor whereas phagocytosis of H37Ra is mediated only through complement receptor [45]. Whether this difference is the major cause for the differential accumulation of avirulent and virulent MTB in individual phagocytic cell is not yet clear. However, it is reasonable to hypothesize that virulent strains once inside the host allow limited accumulation of bacilli by as yet unknown mechanism which is beneficial to the pathogen.

Intracellular bacillary growth is considered to be an indicator of virulence of the MTB strains. Some of the previous studies have suggested that virulent strains grew faster intracellularly and their survival depended on their adaptability inside the host [23], [46], [47], [48]. Our observation that H37Ra infection of THP-1 cells led to a lower bacillary load on day 5 of infection compared to that after infection with virulent strains supported these earlier studies. Avirulent strains fail to sustain inside the host probably due to intracellular killing by the host and induction of apoptosis. According to Sohn *et al*, rapid growth may not essentially represent higher virulence [49]. It has been suggested that drug resistance could confer unfit status on MTB strains [50] which probably was the reason for *in vitro* slow growth of our clinical isolates Beijing, LAM and EAI (resistant to 10, 6 and 8 drugs respectively) compared to that of drug sensitive H37Rv.

Induction of pro-inflammatory cytokine dictates containment of intracellular pathogen, granuloma formation and determines virulence, as well as prevalence of the strain [51]. In a recent study, Portevin *et al* showed a highly significant correlation between the secretion of IL-6 and IL-12 in MTB infected macrophages [20]. In our study secretion of TNF-α and IL-1β were also positively correlated with IL-6 in addition to IL-12, after 24 hours of infection. Our observations also suggested that secretion of proinflammatory cytokines was more likely to be genotype dependent than related to intracellular growth. This was evident from the higher proinflammatory cytokine response elicited by ancestral lineage strain (EAI-5) compared to that by modern lineages (LAM & Beijing) [20]. It has been reported that Beijing genotype consistently induced low levels of proinflammatory cytokines, IL-10 and IL-6 as compared to those induced by H37Rv and other genotypes [16], [18], [49], [52]. The ability of the strains from Beijing genotype to suppress the proinflammatory protective cytokines like TNF-α may be a key to their success as the most virulent genotype [17], [40]. In addition, Beijing strains produce a unique phenolic glycolipid that abolishes the host’s ability to control the infection [53]. Analogous to these findings, we have observed significantly lower induction of proinflammatory cytokine response by our Beijing strain and higher response for ancient EAI strain. EAI-5, used in the present study was drug resistant, though majority of EAI strains in our earlier spoligotyping study were drug sensitive [54]. Further, both LAM-6 and H37Rv belonging to lineage 4, showed similar proinflammatory response. Thus, all the clinical isolates showed patterns of immune response typical for their respective genotypes with no deviations due to drug resistance.

Infection of human alveolar macrophages by MTB has been reported to be sufficient to induce classical apoptosis by extrinsic pathway mediated by TNF-α in an autocrine/paracrine manner and proinflammatory cytokines directly or indirectly modulated apoptotic response depending on the degree of virulence of the strain [55]. In the present study too, we observed a strain specific apoptotic response that correlated well with proinflammatory cytokine induction by only the virulent strains. In the only study reporting the relationship between phagocytosis and apoptosis in MTB infected host, phagocytic index for ‘>20 bacilli/cell’ showed a positive correlation and that for ‘1–10 bacilli/cell’ was negatively correlated with apoptosis [23]. Whereas, in the present study, apoptosis showed a positive and negative correlation with phagocytic index for ‘>10 bacilli/cell’ and 1–5 bacilli/cell’ respectively. It was evident that proinflammatory cytokines and higher initial accumulation of bacilli inside the host cell were the two determining factors for host cell apoptosis.

Recently a similar study [44] assessing a lineage specific response and intracellular growth of MTB strains was published while our manuscript was under preparation. However, to our knowledge, ours is the first report in which all the important parameters like intracellular growth, phagocytic index, induction of cytokines and apoptosis have been monitored and correlated for appropriately characterized MTB lineages from India. It is also unique, as it involved the drug resistant strains from different lineages and for the first time illustrated that the high and low inflammatory responses induced by ancient and modern lineages respectively, were not influenced by their drug resistant status. Such responses may reflect the differential survival strategies employed by clinical isolates to subvert the host immunity. These observations will pave way for better understanding of differential immunopathological interactions involving different genotypes in tuberculosis.

## Supporting Information

Figure S1 **Genetic diversity of** ***M. tuberculosis*** **clinical isolates studied.** Neighbour-joining tree, based on 12 loci MIRU-VNTR typing and 43 spacer spoligotyping showing the phylogenetic relationship of strains in present study along with186 reference strains of MTB complex.
(PDF)

## References

[1] WHO Tuberculosis Global Factsheet (2012) World Health Organization. Available: http://www.who.int/mediacentre/factsheets/fs104/en/.

[2] Russell DG (2007) Who puts the tubercle in tuberculosis? Nat Rev Microbiol 5: 39–47. 17160001 10.1038/nrmicro1538

[3] Cooper AM, Khader SA (2008) The role of cytokines in the initiation, expansion, and control of cellular immunity to tuberculosis. Immunol Rev 226: 191–204. 19161425 10.1111/j.1600-065X.2008.00702.x PMC4298252

[4] Thuong NT, Hawn TR, Thwaites GE, Chau TT, Lan NT, et al (2007) A polymorphism in human TLR2 is associated with increased susceptibility to tuberculous meningitis. Genes Immun 8: 422–428. 17554342 10.1038/sj.gene.6364405

[5] Pitchappan RM, Agrewala JN, Dheenadhayalan V, Ivanyi J (1997) Major histocompatibility complex restriction in tuberculosis susceptibility. J Biosci 22: 47–57.

[6] Pravica V, Perrey C, Stevens A, Lee JH, Hutchinson IV (2000) A single nucleotide polymorphism in the first intron of the human IFN-gamma gene: absolute correlation with a polymorphic CA microsatellite marker of high IFN-gamma production. Hum Immunol 61: 863–866. 11053629 10.1016/s0198-8859(00)00167-1

[7] Manca C, Tsenova L, Barry CE, Bergtold A, Freeman S, et al (1999) *Mycobacterium tuberculosis* CDC1551 induces a more vigorous host response in vivo and in vitro, but is not more virulent than other clinical isolates. J Immunol 162: 6740. 10352293

[8] Bosio CM, Gardner D, Elkins KL (2000) Infection of B cell-deficient mice with CDC 1551, a clinical isolate of *Mycobacterium tuberculosis*: delay in dissemination and development of lung pathology. J Immunol 164: 6417. 10843697 10.4049/jimmunol.164.12.6417

[9] Van Embden JD, Cave MD, Crawford JT, Dale JW, Eisenach KD, et al (1993) Strain identification of *Mycobacterium tuberculosis* by DNA fingerprinting: recommendations for a standardized methodology. J Clin Microbiol 31: 406–409. 8381814 10.1128/jcm.31.2.406-409.1993 PMC262774

[10] Supply P, Lesjean S, Savine E, Kremer K, van Soolingen D, et al (2001) Automated high-throughput genotyping for study of global epidemiology of *Mycobacterium tuberculosis* based on mycobacterial interspersed repetitive units. J Clin Microbiol 39: 3563–3571. 11574573 10.1128/JCM.39.10.3563-3571.2001 PMC88389

[11] Comas I, Gagneux S (2009) The past and future of tuberculosis research. PLoS pathog 5: e1000600. 19855821 10.1371/journal.ppat.1000600 PMC2745564

[12] Hoal-van Helden EG, Stanton LA, Warren R, Richardson M, van Helden PD (2001) Diversity of in vitro cytokine responses by human macrophages to infection by *Mycobacterium tuberculosis* strains. Cell Biol Int 25: 83–90. 11237411 10.1006/cbir.2000.0680

[13] Lopez B, Aguilar D, Orozco H, Burger M, Espitia C, et al (2003) A marked difference in pathogenesis and immune response induced by different *Mycobacterium tuberculosis* genotypes. Clin Exp Immunol 133: 30–37. 12823275 10.1046/j.1365-2249.2003.02171.x PMC1808750

[14] Chacón-Salinas R, Serafin Lopez J, Ramos-Payán R, Méndez-Aragón P, Hernández-Pando R, et al (2005) Differential pattern of cytokine expression by macrophages infected in vitro with different *Mycobacterium tuberculosis* genotypes. Clin Exp Immunol 140: 443–449. 15932505 10.1111/j.1365-2249.2005.02797.x PMC1809389

[15] Sinsimer D, Huet G, Manca C, Tsenova L, Koo MS, et al (2008) The phenolic glycolipid of *Mycobacterium tuberculosis* differentially modulates the early host cytokine response but does not in itself confer hypervirulence. Infect Immun 76: 3027–3036. 18443098 10.1128/IAI.01663-07 PMC2446685

[16] Manca C, Reed MB, Freeman S, Mathema B, Kreiswirth B, et al (2004) Differential monocyte activation underlies strain-specific *Mycobacterium tuberculosis* pathogenesis. Infect Immun 72: 5511. 15322056 10.1128/IAI.72.9.5511-5514.2004 PMC517425

[17] Theus SA, Cave MD, Eisenach KD (2005) Intracellular macrophage growth rates and cytokine profiles of *Mycobacterium tuberculosis* strains with different transmission dynamics. J Infect Dis 191: 453–460. 15633105 10.1086/425936

[18] Tanveer M, Hasan Z, Kanji A, Hussain R, Hasan R (2009) Reduced TNF-alpha and IFN-gamma responses to Central Asian strain 1 and Beijing isolates of *Mycobacterium tuberculosis* in comparison with H37Rv strain. Trans R Soc Trop Med Hyg 103: 581–587. 19375139 10.1016/j.trstmh.2009.03.014

[19] Marquina-Castillo B, Garcia-Garcia L, Ponce-de-Leon A, Jimenez-Corona ME, Bobadilla-Del Valle M, et al (2009) Virulence, immunopathology and transmissibility of selected strains of *Mycobacterium tuberculosis* in a murine model. Immunol 128: 123–133. 10.1111/j.1365-2567.2008.03004.x PMC2747145 19191912

[20] Portevin D, Gagneux S, Comas I, Young D (2011) Human macrophage responses to clinical isolates from the *Mycobacterium tuberculosis* complex discriminate between ancient and modern lineages. PLoS pathog 7: e1001307. 21408618 10.1371/journal.ppat.1001307 PMC3048359

[21] Keane J, Remold HG, Kornfeld H (2000) Virulent *Mycobacterium tuberculosis* strains evade apoptosis of infected alveolar macrophages. J Immunol 164: 2016. 10657653 10.4049/jimmunol.164.4.2016

[22] Rojas M, Olivier M, Gros P, Barrera LF, Garcia LF (1999) TNF-alpha and IL-10 modulate the induction of apoptosis by virulent *Mycobacterium tuberculosis* in murine macrophages. J Immunol 162: 6122–6131. 10229855

[23] Rajavelu P, Das SD (2007) A correlation between phagocytosis and apoptosis in THP-1 cells infected with prevalent strains of *Mycobacterium tuberculosis* . Microbiol Immunol 51: 201. 17310088 10.1111/j.1348-0421.2007.tb03902.x

[24] Brudey K, Driscoll J, Rigouts L, Prodinger W, Gori A, et al (2006) *Mycobacterium tuberculosis* complex genetic diversity: mining the fourth international spoligotyping database (SpolDB4) for classification, population genetics and epidemiology. Bmc Microbiol 6: 23. 16519816 10.1186/1471-2180-6-23 PMC1468417

[25] Honore-Bouakline S, Vincensini JP, Giacuzzo V, Lagrange PH, Herrmann JL (2003) Rapid diagnosis of extrapulmonary tuberculosis by PCR: impact of sample preparation and DNA extraction. Journal of clinical microbiology 41: 2323–2329. 12791844 10.1128/JCM.41.6.2323-2329.2003 PMC156509

[26] Brosch R, Gordon SV, Marmiesse M, Brodin P, Buchrieser C, et al (2002) A new evolutionary scenario for the *Mycobacterium tuberculosis* complex. Proc Natl Acad Sci U S A 99: 3684. 11891304 10.1073/pnas.052548299 PMC122584

[27] Wolf AJ, Linas B, Trevejo-Nuñez GJ, Kincaid E, Tamura T, et al (2007) *Mycobacterium tuberculosis* infects dendritic cells with high frequency and impairs their function in vivo. J Immunol 179: 2509–2519. 17675513 10.4049/jimmunol.179.4.2509

[28] Ganatra RD, Buddemeyer EU, Deodhar MN, Narkar AA, Powar DM, et al (1980) Modifications in biphasic liquid-scintillation vial system for radiometry. J Nucl Med 21: 480–483. 6768855

[29] Lambrecht RS, Carriere JF, Collins MT (1988) A model for analyzing growth kinetics of a slowly growing Mycobacterium sp. Appl environ microbiol 54: 910–916. 3377502 10.1128/aem.54.4.910-916.1988 PMC202572

[30] Boeuf P, Vigan-Womas I, Jublot D, Loizon S, Barale JC, et al (2005) CyProQuant-PCR: a real time RT-PCR technique for profiling human cytokines, based on external RNA standards, readily automatable for clinical use. BMC Immunol 6: 5. 15748278 10.1186/1471-2172-6-5 PMC555737

[31] Tripathy NK, Chauhan SK, Nityanand S (2004) Cytokine mRNA repertoire of peripheral blood mononuclear cells in Takayasu’s arteritis. Clin Exp Immunol 138: 369–374. 15498051 10.1111/j.1365-2249.2004.02613.x PMC1809220

[32] Livak KJ, Schmittgen TD (2001) Analysis of relative gene expression data using real-time quantitative PCR and the 2-[Delta][Delta] CT method. Methods 25: 402–408. 11846609 10.1006/meth.2001.1262

[33] Duan L, Gan H, Arm J, Remold HG (2001) Cytosolic phospholipase A2 participates with TNF-α in the induction of apoptosis of human macrophages infected with *Mycobacterium tuberculosis* H37Ra. J Immunol 166: 7469. 11390500 10.4049/jimmunol.166.12.7469

[34] Weniger T, Krawczyk J, Supply P, Niemann S, Harmsen D (2010) MIRU-VNTRplus: a web tool for polyphasic genotyping of *Mycobacterium tuberculosis* complex bacteria. Nucl. Acids Res 38: W326–W331. 10.1093/nar/gkq351 PMC2896200 20457747

[35] Van der Spuy GD, Kremer K, Ndabambi SL, Beyers N, Dunbar R, et al (2009) Changing *Mycobacterium tuberculosis* population highlights clade-specific pathogenic characteristics. Tuberculosis (Edinb) 89: 120–125. 19054717 10.1016/j.tube.2008.09.003

[36] Almeida D, Rodrigues C, Ashavaid TF, Lalvani A, Udwadia ZF, et al (2005) High incidence of the Beijing genotype among multidrug-resistant isolates of *Mycobacterium tuberculosis* in a tertiary care center in Mumbai, India. Clin infect dis 40: 881–886. 15736024 10.1086/427940

[37] Narayanan S, Gagneux S, Hari L, Tsolaki AG, Rajasekhar S, et al (2008) Genomic interrogation of ancestral *Mycobacterium tuberculosis* from south India. Infect Genet Evol 8: 474–483. 18024233 10.1016/j.meegid.2007.09.007 PMC5538575

[38] Bifani PJ, Mathema B, Kurepina NE, Kreiswirth BN (2002) Global dissemination of the *Mycobacterium tuberculosis* W-Beijing family strains. Trends Microbiol 10: 45–52. 11755085 10.1016/s0966-842x(01)02277-6

[39] Lan NTN, Lien HTK, Tung LB, Borgdorff MW, Kremer K, et al (2003) *Mycobacterium tuberculosis* Beijing genotype and risk for treatment failure and relapse, Vietnam. Emerg Infect Dis 9: 1633. 14720411 10.3201/eid0912.030169 PMC3034339

[40] Parwati I, Van Crevel R, Van Soolingen D (2010) Possible underlying mechanisms for successful emergence of the *Mycobacterium tuberculosis* Beijing genotype strains. Lancet Infect Dis 10: 103–111. 20113979 10.1016/S1473-3099(09)70330-5

[41] Lazzarini LCO, Spindola SM, Bang H, Gibson AL, Weisenberg S, et al (2008) RDRio *Mycobacterium tuberculosis* infection is associated with a higher frequency of cavitary pulmonary disease. J clin microbiol 46: 2175–2183. 18463217 10.1128/JCM.00065-08 PMC2446940

[42] Ignatova A, Dubiley S, Stepanshina V, Shemyakin I (2006) Predominance of multi-drug-resistant LAM and Beijing family strains among *Mycobacterium tuberculosis* isolates recovered from prison inmates in Tula Region, Russia. J med microbiol 55: 1413. 17005791 10.1099/jmm.0.46575-0

[43] Torrelles JB, Knaup R, Kolareth A, Slepushkina T, Kaufman TM, et al (2008) Identification of *Mycobacterium tuberculosis* clinical isolates with altered phagocytosis by human macrophages due to a truncated lipoarabinomannan. J Biol Chem 283: 31417–31428. 18784076 10.1074/jbc.M806350200 PMC2581576

[44] Sarkar R, Lenders L, Wilkinson KA, Wilkinson RJ, Nicol MP (1212) Modern Lineages of *Mycobacterium tuberculosis* Exhibit Lineage-Specific Patterns of Growth and Cytokine Induction in Human Monocyte-Derived Macrophages. PLoS One 7: e43170. 10.1371/journal.pone.0043170 PMC3420893 22916219

[45] Schlesinger LS (1993) Macrophage phagocytosis of virulent but not attenuated strains of *Mycobacterium tuberculosis* is mediated by mannose receptors in addition to complement receptors. J Immunol 150: 2920–2930. 8454864

[46] Park JS, Tamayo MH, Gonzalez-Juarrero M, Orme IM, Ordway DJ (2006) Virulent clinical isolates of *Mycobacterium tuberculosis* grow rapidly and induce cellular necrosis but minimal apoptosis in murine macrophages. J Leukoc Biol 79: 80–86. 16275894 10.1189/jlb.0505250

[47] Silver RF, Li Q, Ellner JJ (1998) Expression of Virulence of *Mycobacterium tuberculosis*within Human Monocytes: Virulence Correlates with Intracellular Growth and Induction of Tumor Necrosis Factor Alpha but Not with Evasion of Lymphocyte-Dependent Monocyte Effector Functions. Infect Immun 66: 1190–1199. 9488413 10.1128/iai.66.3.1190-1199.1998 PMC108033

[48] Zhang M, Gong J, Lin Y, Barnes PF (1998) Growth of virulent and avirulent *Mycobacterium tuberculosis* strains in human macrophages. Infect Immun 66: 794–799. 9453643 10.1128/iai.66.2.794-799.1998 PMC107972

[49] Sohn H, Lee KS, Kim SY, Shin DM, Shin SJ, et al (2009) Induction of cell death in human macrophages by a highly virulent Korean isolate of *Mycobacterium tuberculosis* and the virulent strain H37Rv. Scand J Immunol 69: 43–50. 19140876 10.1111/j.1365-3083.2008.02188.x

[50] Von Groll A, Martin A, Stehr M, Singh M, Portaels F, et al (2010) Fitness of *Mycobacterium tuberculosis* strains of the W-Beijing and Non-W-Beijing genotype. PLoS One 5: e10191. 20419138 10.1371/journal.pone.0010191 PMC2855714

[51] Freeman S, Post FA, Bekker LG, Harbacheuski R, Steyn LM, et al (2006) *Mycobacterium tuberculosis* H37Ra and H37Rv differential growth and cytokine/chemokine induction in murine macrophages in vitro. J interferon cytokine res 26: 27–33. 16426145 10.1089/jir.2006.26.27

[52] Lasunskaia E, Ribeiro S, Manicheva O, Gomes LL, Suffys PN, et al (2010) Emerging multidrug resistant *Mycobacterium tuberculosis* strains of the Beijing genotype circulating in Russia express a pattern of biological properties associated with enhanced virulence. Microbes and Infection 12: 467–475. 20215000 10.1016/j.micinf.2010.02.008

[53] Domenech P, Reed MB (2009) Rapid and spontaneous loss of phthiocerol dimycocerosate (PDIM) from *Mycobacterium tuberculosis* grown in vitro: implications for virulence studies. Microbiol 155: 3532. 10.1099/mic.0.029199-0 PMC5154741 19661177

[54] Kulkarni S, Sola C, Filliol I, Rastogi N, Kadival G (2005) Spoligotyping of *Mycobacterium tuberculosis* isolates from patients with pulmonary tuberculosis in Mumbai, India. Res microbiol 156: 588–596. 15862459 10.1016/j.resmic.2005.01.005

[55] Ciaramella A, Cavone A, Santucci MB, Amicosante M, Martino A, et al. (2002) Proinflammatory cytokines in the course of *Mycobacterium tuberculosis*–induced apoptosis in monocytes/macrophages. J Infect Dis 186: 1277. 10.1086/344645 12402197

