# Supplementary figures and images for: Drug Resistant Clinical Isolates of Mycobacterium tuberculosis from Different Genotypes Exhibit Differential Host Responses in THP-1 Cells
Source: PLoS One. 2013 May 7;8(5):e62966. doi: 10.1371/journal.pone.0062966 (PMC3646887; doi:10.1371/journal.pone.0062966)

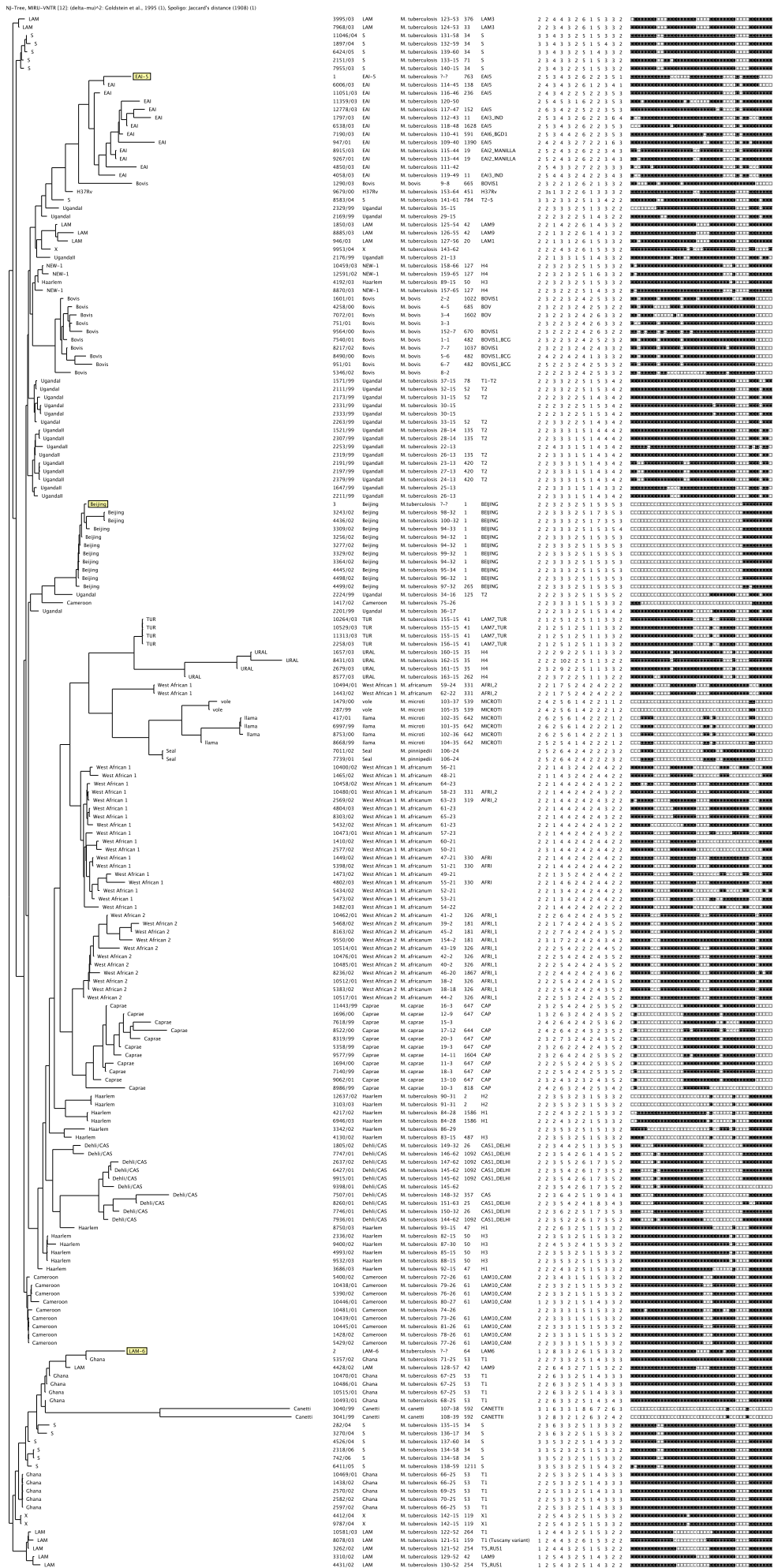

Supplement: Figure S1 — Genetic diversity of M. tuberculosis clinical isolates studied. Neighbour-joining tree, based on 12 loci MIRU-VNTR typing and 43 spacer spoligotyping showing the phylogenetic relationship of strains in present study along with186 reference strains of MTB complex. (PDF) [file pone.0062966.s001.pdf]
